# Design Guidelines for Improving Mobile Sensing Data Collection: Prospective Mixed Methods Study

**DOI:** 10.2196/55694

**Published:** 2024-11-18

**Authors:** Christopher Slade, Roberto M Benzo, Peter Washington

**Affiliations:** 1 Computer Science Department Brigham Young University—Hawaii Laie, HI United States; 2 Information and Computer Sciences Department University of Hawaii at Manoa Honolulu, HI United States; 3 Division of Cancer Prevention & Control Department of Internal Medicine, Wexner Medical Center Ohio State University Columbus, OH United States; 4 Arthur G James Cancer Hospital The Ohio State University Comprehensive Cancer Center Columbus, OH United States

**Keywords:** mobile health sensing, mHealth, active data collection, passive data collection, ecological momentary assessment, mobile data, mobile phone, machine learning, real-world setting, mixed method, college, student, user data, data consistency

## Abstract

**Background:**

Machine learning models often use passively recorded sensor data streams as inputs to train machine learning models that predict outcomes captured through ecological momentary assessments (EMA). Despite the growth of mobile data collection, challenges in obtaining proper authorization to send notifications, receive background events, and perform background tasks persist.

**Objective:**

We investigated challenges faced by mobile sensing apps in real-world settings in order to develop design guidelines. For active data, we compared 2 prompting strategies: setup prompting, where the app requests authorization during its initial run, and contextual prompting, where authorization is requested when an event or notification occurs. Additionally, we evaluated 2 passive data collection paradigms: collection during scheduled background tasks and persistent reminders that trigger passive data collection. We investigated the following research questions (RQs): (RQ1) how do setup prompting and contextual prompting affect scheduled notification delivery and the response rate of notification-initiated EMA? (RQ2) Which authorization paradigm, setup or contextual prompting, is more successful in leading users to grant authorization to receive background events? and (RQ3) Which polling-based method, persistent reminders or scheduled background tasks, completes more background sessions?

**Methods:**

We developed mobile sensing apps for iOS and Android devices and tested them through a 30-day user study asking college students (n=145) about their stress levels. Participants responded to a daily EMA question to test active data collection. The sensing apps collected background location events, polled for passive data with persistent reminders, and scheduled background tasks to test passive data collection.

**Results:**

For RQ1, setup and contextual prompting yielded no significant difference (ANOVA *F*_1,144_=0.0227; *P*=.88) in EMA compliance, with an average of 23.4 (SD 7.36) out of 30 assessments completed. However, qualitative analysis revealed that contextual prompting on iOS devices resulted in inconsistent notification deliveries. For RQ2, contextual prompting for background events was 55.5% (*χ*^2^_1_=4.4; *P*=.04) more effective in gaining authorization. For RQ3, users demonstrated resistance to installing the persistent reminder, but when installed, the persistent reminder performed 226.5% more background sessions than traditional background tasks.

**Conclusions:**

We developed design guidelines for improving mobile sensing on consumer mobile devices based on our qualitative and quantitative results. Our qualitative results demonstrated that contextual prompts on iOS devices resulted in inconsistent notification deliveries, unlike setup prompting on Android devices. We therefore recommend using setup prompting for EMA when possible. We found that contextual prompting is more efficient for authorizing background events. We therefore recommend using contextual prompting for passive sensing. Finally, we conclude that developing a persistent reminder and requiring participants to install it provides an additional way to poll for sensor and user data and could improve data collection to support adaptive interventions powered by machine learning.

## Introduction

**Figure 1:**
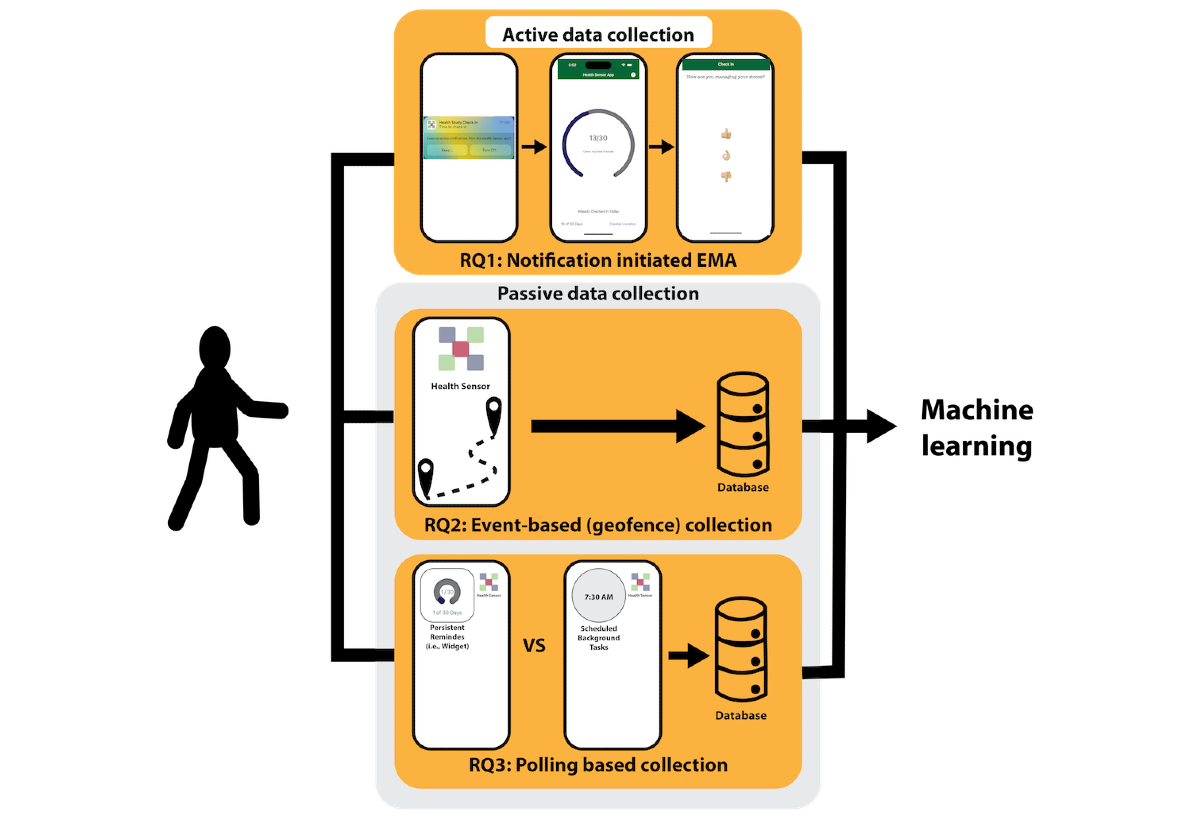
Machine learning models often use passively recorded sensor streams as inputs to predict outcomes captured through EMA. We explore the feasibility of collecting both passive and active data on consumer mobile devices to answer the following research questions (RQs): (RQ1) how do contextual prompting and setup prompting affect scheduled notification delivery and the response rate of notification-initiated EMA? (RQ2) Which authorization paradigm, setup or contextual prompting, is more successful in leading users to grant authorization to receive background events? and (RQ3) Which polling-based method, persistent reminders or scheduled background tasks, completes more background sessions? EMA: ecological momentary assessment.

Mobile and ubiquitous devices are valuable tools for gathering patient-generated health data in real-world settings [1-11]. Prior to the advent of the smartphone, mobile devices were predominantly used to collect data actively, usually through ecological momentary assessments (EMA). EMA involves “repeated sampling of participants’ current behaviors and experiences in real-time in the participants’ natural environment” [12-14]. EMA can include completing journals, diaries, and survey questions [15], providing audio or video samples [16,17], or participating in digital or physical tests [18]. As mobile devices evolved to include more sensors and access to health data, mobile apps started to use passive data collection methods [19-22], where mobile devices collect data without involving the end user. Today, machine learning models often use passively recorded sensor data streams as inputs to machine learning models that predict health outcomes or events captured through EMA [23-29] (Figure 1).

Despite the growth of mobile data collection in health research, several challenges for mobile sensing on consumer mobile devices persist. These challenges are due to implementation decisions made by the developers of major mobile devices (ie, iOS or iPhone and Android) to protect users’ privacy, preserve battery life, and minimize distractions. Passive data collection requires access to private user data, whereas active data collection needs to interrupt users to initiate an EMA. In this work, we studied how various user interface (UI) decisions for obtaining authorization at the app level affect the success of both active and passive mobile sensing. Specifically, we tested the following authorization scenarios: (1) users explicitly granting authorization to receive notifications to initiate EMA, (2) users explicitly granting authorization to access background events, and (3) the system implicitly granting background runtime to collect data through polling.

The specific authorization procedure for the first 2 scenarios varies depending on the device. An Android device obtains authorization through setup prompting, where the user is prompted during the initial app launch. On the other hand, iOS devices use contextual prompting, where the user is prompted when the first event or notification occurs.

Background runtime for passive sensing is often obtained implicitly. Android and iOS systems determine when to run background tasks based on user actions and battery status. Another way to obtain background runtime is through a persistent reminder. A persistent reminder is a permanent UI feature, like a home screen widget or persistent notification, that receives background runtime to update its UI.

To explore these active and passive sensing implementation scenarios, we developed mobile sensing apps for Android and iOS devices that logged both passive and active data. We tested our apps with a user study to answer the following research questions (RQs; Figure 1): (RQ1) how do contextual prompting and setup prompting affect scheduled notification delivery and the response rate of notification-initiated EMA? We hypothesize that contextual prompting will lead to better EMA compliance because participants will not have to respond to a setup prompt and will have more context when authorizing notifications; (RQ2) which authorization paradigm, setup or contextual prompting, is more successful in leading users to grant authorization to receive background events? We hypothesize that the contextual prompts will improve background event authorization because the added context will help participants understand and feel safe approving the permission prompt; and (RQ3) which polling-based method, persistent reminders or scheduled background tasks, completes more background sessions? We hypothesize that persistent reminders will poll for data more often than background tasks because the mobile operating system (OS) is willing to expend resources to keep the UI up-to-date.

## Methods

### Overview

This section is organized as follows. First, we introduce the authorization procedures that can affect the success of mobile sensing studies. Next, we describe how we used these procedures in our mobile sensing app. Finally, we describe a user study we performed to answer our RQs. A summary of the authorization methods used for each RQ is found in Table 1.

**Table 1:** Summary of authorization methods used in each research question (RQ).

Condition		Authorization method
**RQ1^a^**		
	Android	Setup prompting for notifications
	iOS	Contextual prompting for notifications
**RQ2^b^**		
	Android	Setup prompting for background events
	iOS	Contextual prompting for background events
**RQ3^c^**		
	Background tasks	User actions, device status implies consent
	Persistent reminders	Installation implies consent

^a^RQ1 compares authorization methods for notification-initiated ecological momentary assessment.

^b^RQ2 compares authorization methods for event-driven data collection.

^c^RQ3 compares polling-based collection methods.

### Authorization Procedures

For both Android and iOS, mobile apps must gain explicit authorization before sending notifications or receiving events. Explicit authorization is when a user grants authorization through a permission prompt. At the time of writing, Android devices use setup prompting for explicit authorization, where the user is prompted to grant authorization during setup. The setup prompt can be displayed during the initial launch or when the feature requiring authorization is enabled. iOS devices, on the other hand, use contextual prompting for explicit authorization, where the user is prompted when the first event or notification occurs.

Contextual prompting provides the user with more context to enable a more informed decision. For example, with setup prompting, the user is asked for background location access after consenting, not knowing when or where the location will be accessed. With contextual prompting, the user is prompted when the location is first accessed, helping them understand when their location is accessed. Providing this additional context could help the users feel more comfortable with granting permissions, knowing that the location will be accessed only at a specific location. With setup prompting, on the other hand, we hypothesize that the user might be overwhelmed during setup and deny the request for location or notifications.

While explicit authorization procedures are required for notifications, implicit authorization is needed to grant the background runtime for sensing apps to automatically collect passive sensor and use data. Implicit authorization is when the mobile OS implies authorization based on user actions and device status. Gaining background runtime for scheduled tasks depends on user actions such as app use, swiping an app out of the recent app switcher, and enabling power-saving mode. Device status indicators, such as the battery level, charging state, and network connectivity, are additional factors determining when background runtime is granted. iOS devices always use implicit authorization to determine when to grant runtime. Android devices vary by model and version. However, modern devices include the “adaptive battery” [30] feature, which implicitly grants background runtime.

Installing a permanent UI feature, or persistent reminder, is another way to gain background runtime through implicit authorization. A persistent reminder is part of an app that is always displayed on the mobile device’s home screen, lock screen, or notification center. A persistent reminder gains background runtime to keep its UI updated. In this case, the installation of the persistent reminder implicitly grants authorization to run in the background. The persistent reminder can also remind the participant to complete EMA tasks.

### Mobile Sensing Apps

**Figure 2:**
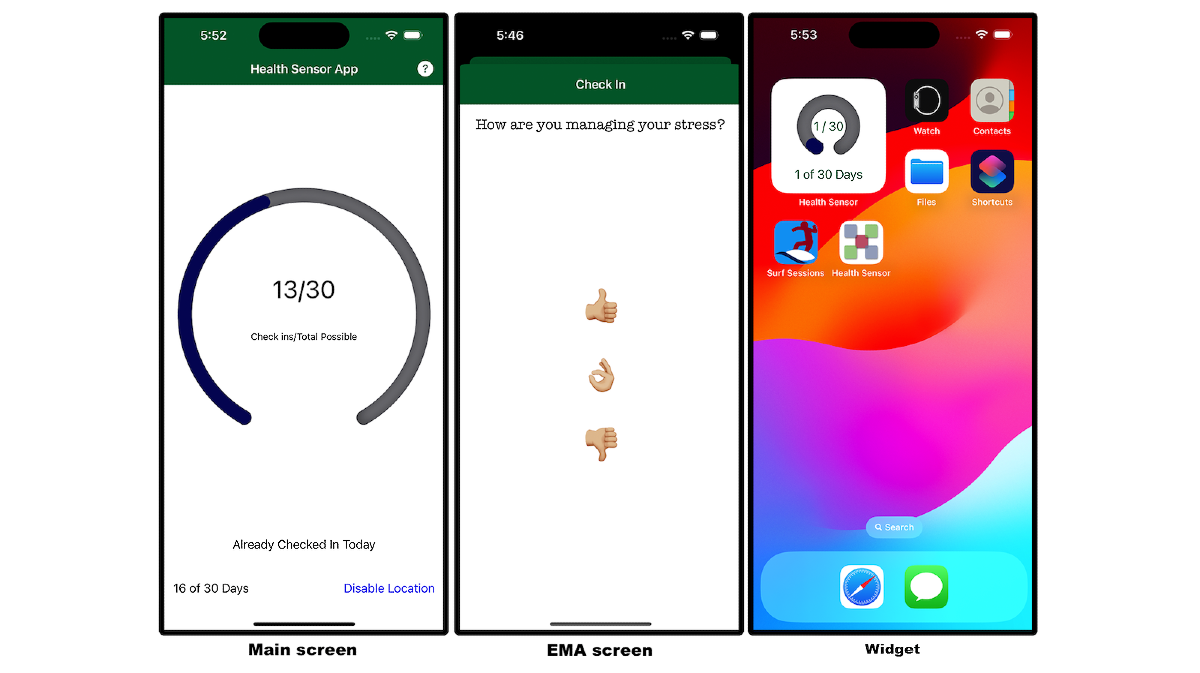
Mobile sensing app. Left: the main screen displays the study progress. Middle: the EMA screen, which asks participants about their stress levels. Participants were asked to complete one assessment per day. Right: the home screen widget is used as a persistent reminder. EMA: ecological momentary assessment.

We developed a mobile sensing app for iOS and Android devices designed to collect active data through EMA and passive data through polling-based and event-based data collection. The native languages of each OS, Swift for iOS and Kotlin for Android, were used for development. Besides native UI differences and authorization procedures outlined above, iOS and Android apps were designed to appear and function identically. The apps featured a home screen widget displaying the study’s progress and serving as a persistent reminder. The main screen, EMA screen, and home screen widget are shown in Figure 2.

### Development Process

We first developed the iOS app and then developed the Android app to match the look and functionality of the iOS app. We tested the apps simultaneously to ensure that Android and iOS devices reported the same data and functioned similarly. We ensured they reported the same number of location events, sent notifications at the same time, and otherwise behaved similarly.

**Figure 3:**
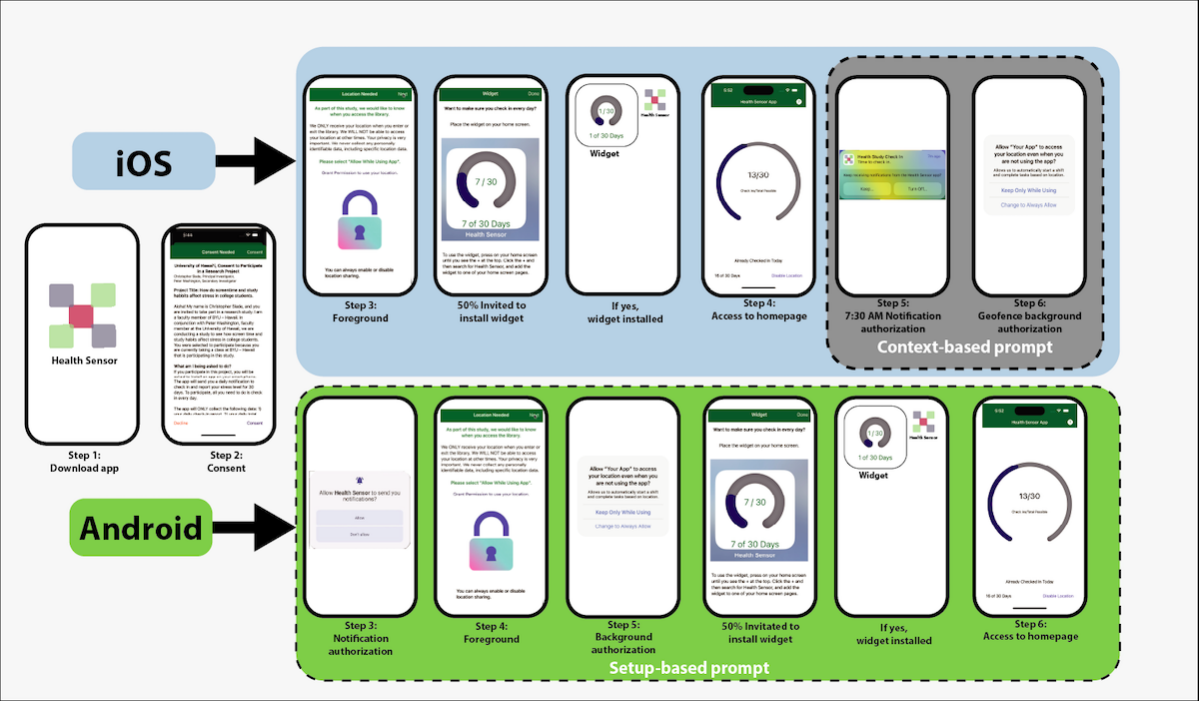
Overview of the difference between authorization procedures on Android and iOS devices. On iOS devices, both notification and background location authorization prompts are received in context. The notification authorization prompt is presented with the first EMA reminder notification, and the background location authorization is presented when the first location event occurs. For Android devices, those prompts are present during the initial launch of the app. EMA: ecological momentary assessment.

The authorization methods we used in this study reflect each mobile OS’ preferred method for gaining authorization. At the time of writing, Android devices do not support contextual prompts for notifications or background events. iOS does not directly support setup prompting for background events and discourages setup prompting for notifications. We chose the OS’ preferred authorization method because users should be familiar with the procedure. Figure 3 highlights the authorization differences.

We originally planned to upload all data to secure storage through the background task. However, during testing, we noticed an inconsistency in uploading the data using only the background task. Thus, we also synced data whenever the app was launched in the foreground. To ensure all data were collected, the collection was confirmed before presenting a “Study Finished” screen along with instructions to take a screenshot.

### RQ1: Notification-Initiated EMA

**Figure 4:**
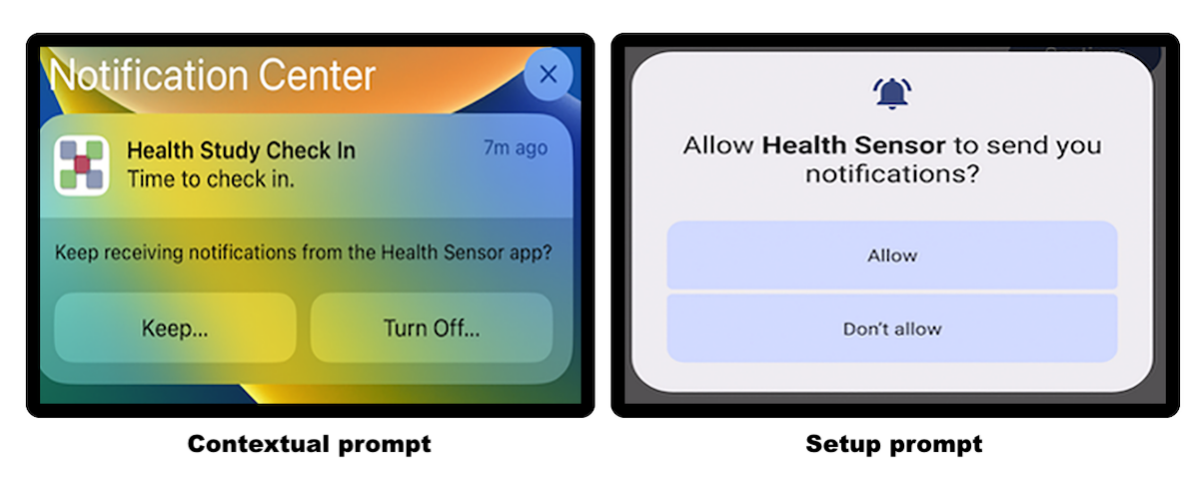
Notification permission prompts. Left: contextual prompt on an iOS device. Right: setup prompt on an Android device. With setup prompting, users are asked to approve notifications during the initial run of the app. Using contextual prompting, users are asked to approve notifications when the first notification arrives.

RQ1 compares setup and contextual prompting for notification authorization to measure their effect on EMA compliance. We compared EMA compliance using the iOS notification system, which uses contextual prompting, against Android’s notification system, which uses setup prompting. To answer RQ1, the apps used notification-initiated EMA to collect active data. We performed a simple single-question EMA once per day, for which both Android and iOS apps reminded users to complete their EMA through a notification. The Android setup prompt and iOS contextual prompt are displayed in Figure 4.

Notification systems on Android and iOS have other features that could affect compliance. iOS 15 introduced “Focus” times [31], which implements notification deferral, and notification summaries [32]. Android devices also implement notification deferral through a focus mode [33], pausing notifications from selected apps when activated. These features might reduce EMA compliance [34,35].

### RQ2: Event-Based Passive Data Collection

RQ2 explores how setup and contextual prompting affect the success of event-based passive data collection. In the event-based collection, an event, such as a location change, phone call, message, or health alert, initiates the data collection. Sensing apps need explicit authorization to receive background events. Our apps used location changes as the event of interest and monitored a geofence, or circular region, where participants frequently entered and exited, triggering a location event. iOS devices used contextual prompting to authorize receipt of background location events, and Android devices used setup prompting.

### RQ3: Polling-Based Passive Data Collection

RQ3 explores the ability of traditional background tasks and persistent reminders to collect data passively. Both Android and iOS apps implemented and scheduled a daily background task. The background task logged the time and uploaded all logs to cloud storage. We also uploaded the data upon app launch to ensure that all data were collected. For persistent reminders, we implemented a home screen widget, shown in Figure 2, and requested a daily refresh, which was also logged.

We explored users’ willingness to install the persistent reminder. Each participant had an equal chance of being assigned to either (1) a control group that did not receive prompts or notifications to install the widget or (2) an experimental group that did receive prompts and notifications. One group of participants was provided verbal instructions for installing the widget.

### Alternative Study Designs

We used the mobile OS’ default or preferred authorization procedures to test their effectiveness in the wild, similar to other studies that test the feasibility of cross-platform mobile sensing [36-38]. The strength of this approach is that it does not limit the study to users of one particular device (iOS or Android), increasing the diversity of the participants. It also works to understand mobile sensing in the wild, revealing insights into potential pitfalls while developing cross-platform mobile sensing frameworks. However, this limits our ability to isolate study variables because other differences between the mobile OS implementations or device hardware could impact our results.

Studies focusing on a single mobile OS sometimes sacrifice user diversity in favor of isolating variables. These studies are favored when testing new features [39,40] or focusing on specific variables [41]. Android tends to be used in these studies due to its more open programming interface.

### User Study

We tested our sensing apps through a user study that aimed to identify the impact of screen time and study time on college students’ stress levels. Participants installed our apps on their mobile devices and participated in our study for 30 days. To test event-based collection, a geofence that contained the school library and classrooms was used to calculate study time. Background tasks purported to collect screen time statistics to test polling-based collection.

Active data was collected through a simple EMA that asked participants how they managed their stress levels, as shown in Figure 2. A notification was sent each morning at 7:30 AM to remind users to complete their EMA. Students could complete their daily EMA until the end of the day (midnight). Participants could respond with a thumbs up, thumbs down, or neutral.

### Ethical Considerations

The University of Hawaii Institutional Review Board approved the study under protocol #2022-0722, and the Brigham Young University—Hawaii Institutional Review Board approved it under protocol #22-72. All participants consented to participate in the study through the sensing app and received extra credit in their courses for participating. The following measures were implemented to ensure user privacy and participant safety: (1) after installation, the mobile app required the participants to electronically consent before collecting any data; (2) students who did not want to participate were given an alternative extra credit assignment, representing the same time commitment as completing the study; (3) the app anonymized all data collected before being uploaded to cloud storage, so the instructors could not identify the participants’ data, including their study habits; (4) follow-up paper surveys were collected by a volunteer, not the class instructor; and (5) at the end of the study, students were instructed by the app to submit a screenshot to their courses’ learning management system, and all students were provided with the same amount of extra credit regardless of EMA compliance or the amount of data collected.

### Recruitment

We recruited 145 college students in Computer Science, Information Technology, Business, and Science classes at Brigham Young University—Hawaii. The students were offered extra credit incentives to install our app on their mobile devices and actively engage in the study for 30 days.

To ensure students that their professors could not identify their study habits, specific demographic information was not collected, and the app anonymized all data before uploading logs to cloud storage. However, the general demographics of the recruitment base included college students aged 18-26 years, with around 60% being female and 40% being male. Students represented a variety of races and cultures, with a distribution of about 40% White or non-Hispanic, 15% Native Hawaiian and Other Pacific Islanders, 25% Asian, 15% two or more races, and 5% other.

### Exit Survey

Upon completion of the study, a follow-up survey was provided to participants, which 48 participants completed. The survey was provided on paper during the course’s final exam. To ensure privacy, a volunteer, not the instructor, distributed and collected the survey from participants. To preserve privacy, we did not correlate the survey with the data collected by the mobile app. The survey allowed us to gather qualitative insights regarding the differing success rates across the authorization paradigms. The participants were asked (1) what reminded them to complete their assessment, (2) their general thoughts on granting background location access, (3) whether they knew about and installed the home screen widget and their thoughts on installing a widget, and (4) suggestions to improve the app.

## Results

### Data Cleaning and Analysis

We initially logged 178 (126 iOS and 52 Android) users who installed the app and consented to participate in the study. We removed users from the study if the logs showed they participated for fewer than 5 days, which meant that they deleted the app fewer than 5 days after starting. We did not detect any users who deleted the app after participating for 5 days. This yielded 145 participants, representing 105 iOS and 40 Android users. Each participant had an equal chance of being assigned to the widget or control group by the app. A total of 52 individuals (34 iOS and 18 Android) were randomly assigned to the widget group, where the app prompted them to install the widget. In total, 82 participants were randomly assigned to the control group, which did not receive the prompts, and 11 participants (8 iOS and 3 Android) received verbal instructions to install the widget on their home screens.

To conduct our qualitative analysis, we coded responses to the survey based on general patterns that emerged in at least five responses. The questions and the number of themed responses are listed in Table 2.

**Table 2:** Summary of exit survey responses for each question, separated by device. At the end of the study, participants were administered a paper survey to complete during their final exam, of which 48 participants (33 iOS and 15 Android) completed. Responses were coded based on general themes that emerged in at least 5 responses. Themes with fewer than 5 responses are not listed.

		iOS (n=33), n (%)	Android (n=15), n (%)
**Question 1: What reminded you to check-in?**			
	Notifications	12 (36)	9 (60)
	Set alarm	9 (27)	0 (0)
	Widget	5 (15)	0 (0)
	Saw app	5 (15)	3 (20)
**Question 2: Did you allow the app to use your location? Why or why not?**			
	Yes	25 (76)	15 (100)
	No	2 (6)^a^	0 (0)
	It would help the study	7 (21)	6 (40)
	It was required	4 (12)	3 (20)
	Safe	4 (12)	1 (7)
**Question 3: Did you install the widget? Why or why not?**			
	Yes	15 (45)	2 (13)
	No	15 (45)	13 (87)
	Did not know about it	7 (21)	4 (27)
	Helped me remember to check-in	6 (18)	1 (7)
	Did not want to change the home screen or do not use widgets	4 (12)	2 (13)
**Question 4: Do you have any comments or suggestions on improving the app?**			
	Better notifications	15 (45)	3 (20)
	Select notification time	4 (12)	2 (13)

^a^1 worried about tracking.

### RQ1: Notification-Initiated EMA

To answer RQ1, we compared notification authorization procedures: contextual prompting implemented in iOS versus setup prompting implemented in Android by measuring the number of completed EMA. On average, participants across all conditions completed 23.4 (SD 7.38) assessments out of 30. iPhone users completed 23.46 (SD 7.02) assessments, and Android users completed 23.25 (SD 7.9) assessments on average. We observed no statistically significant difference in the number of completed assessments when examining device-specific differences as shown by an ANOVA test (*F*_1,144_=0.0227; *P*=.88) or when comparing the widget group to the control group (*F*_1,144_=1.33; *P*=.27).

Although our quantitative results demonstrated no difference between devices, our qualitative results indicated that contextual prompting, notification deferral, and notification summaries on iOS devices affected notification delivery. Nine Android users reported that notifications were the primary method of reminding them to complete their EMA. Despite 9 iOS users stating that the notifications helped them remember to complete the task, 15 iOS users surveyed said the notifications did not appear consistently. Nine participants even set their alarms or reminders. For example, P32 stated, “Notifications weren’t working, so I had an event in my calendar to remind me.”

### RQ2: Event-Driven Passive Data Collection Results

In RQ2, we compared the effectiveness of contextual prompting (iOS) and setup prompting (Android) in gaining authorization to access background location events. A total of 28% (11/40) of Android and 49% (51/105) of iOS users enabled background location permission. Contextual and setup prompting differed significantly (*χ*^2^_1_=4.4; *P*=04). Thus, contextual prompting was 55.5% more effective than setup prompting in gaining authorization for background location events.

Our poststudy survey asked participants about their willingness to share their location with the sensing app. A total of 40 surveyed participants said they authorized background location access versus 2 participants who reported not sharing their location. Only one participant, P10 (iOS), expressed apprehension about sharing their location, stating, “I was afraid it could track me.” However, 13 participants surveyed expressed a willingness to share their location to contribute to the study’s objectives and thought they granted the needed authorization to access background location events. P46 (Android) exemplified this sentiment: “Yes, if it helps the study then I don’t really mind if it has my location.” User’s willingness to share location data for nonresearch reasons will most likely vary, so we emphasize that these results only apply to the context of research studies.

### RQ3: Background Tasks and Persistent Reminders

RQ3 compared traditional background tasks to persistent reminders for polling-based data collection. First, we present our results on how willing users were to install the persistent reminder, and then we present results comparing persistent reminder refreshes to background tasks completed. Finally, we compare background tasks completed by device type and present our qualitative results.

We studied how willing participants were to install the persistent reminder by randomly assigning users to an experimental group where they were notified by the app to install the widget and a control group that did not receive such prompts. In addition, a subgroup of 11 users were given verbal instructions to install the widget. Of 52 participants in the widget group, only 18 (35%) participants installed the home screen widget, with 16 being iOS users and 2 being Android users. One iOS participant in the control group installed the widget. Of the 11 participants who received verbal instructions to install the widget, 5 (46%) participants complied accordingly, of whom 4 were iOS users and 1 was an Android user.

Limiting our data to only users who installed the persistent reminder, on average, the persistent reminder was refreshed 61.2 (SD 16.7) times throughout the study, and the devices performed an average of 7.2 (SD 12.5) background tasks. The two Android devices performed 23 and 42 widget refreshes, with 8 and 6 background tasks, respectively. When users installed the widget, persistent reminders refreshed 266.5% more than background tasks were executed.

Focusing on the difference between Android and iOS, we also observed a significant difference in background tasks completed when comparing devices. iOS devices completed 7.73 (SD 16.32) background sessions during the study. Android devices completed 25.7 (SD 13.96) background sessions. Using data from all participants, an ANOVA test revealed a significant difference in the total number of background sessions between iOS devices and Android devices (*F*_1,144_=37.8; *P*<.001), showing that Android devices were more permissive in granting background runtime. Most iOS devices completed fewer than 10 background sessions, and many did not execute a single background task. In addition, almost half of the Android devices did not complete a daily background task. This demonstrates our difficulty in consistently gaining background runtime, especially on iOS devices.

Our qualitative analysis showed mixed results regarding the installation of the home screen widget. Several participants indicated that the widget helped them remember to complete their daily assessment, as expressed by seven participants. For example, P37 (iOS) stated, “The widget helped me to remember to check in every day and what the study was about.” However, 6 users opted not to use the widget due to concerns about modifying their home screen or preferring not to use widgets. P22 (iOS) mentioned, “My home screen was already full and organized,” while P35 (Android) stated, “I just don’t want to change my home screen.” iOS users may be more willing to add the widget to their home screen because iOS allows for widget stacking, a feature not found in most Android devices. This functionality enables the rotation of different widgets within a stack, eliminating the need to reorganize the home screen to install the widget.

### Effects of Stress Level

Because students experiencing a high stress level might be more motivated to authorize data collection and complete assessments, we divided the participants into 2 groups: a low-stress group (n=117) whose EMA responses averaged better than neutral and a high-stress group (n=28) whose EMA responses averaged lower than neutral. We then compared the two groups’ authorization and data collection rates. There was no statistical difference between the groups in the number of EMA responses (*F*_1,144_=0.928; *P*=.34), the number of background tasks executed (*F*_1,144_=1.54; *P*=.22), the number of participants that enabled location (*χ*^2^_1_=0.4; *P*=.52), or whether they installed the widget (*χ*^2^_1_=0.5; *P*=.57). The stress level of the participants did not significantly impact their willingness to grant authorization, provide EMA responses, or install the widget.

## Discussion

Based on our results, we present design principles to enhance the success of mobile passive-sensing research studies.

### Notification-Initiated EMA

Setup prompts should generally be used for notifications. Although our quantitative results showed no difference between the iOS and Android notification systems, our qualitative analysis revealed that many iOS users did not consistently receive notifications to remind them to complete their EMA. Nine iOS users reported setting their alarms or calendar reminders to remember to complete their EMA. We conjecture that if this study had required participants to complete multiple tasks per day or to follow a strict time frame for EMA tasks, then EMA compliance on iOS devices would have suffered.

We hypothesized that contextual prompts would work more effectively. However, they lose their context when notification deferrals and summaries are enabled. Only users who searched through their notification summaries would have found the contextual prompt and authorized future notifications. With setup prompting, by contrast, users authorize the notifications immediately after consenting.

Our qualitative results show that notification deferral and notification summaries can cause delayed and missed responses to EMA notifications on iOS devices. New methods should be developed to help participants remember to complete their assessments in a timely manner. iOS allows developers to mark notifications as time-sensitive, which increases their visibility to the user and should be used for time-sensitive EMA. This study did not use time-sensitive notifications because our assessment did not require a time-sensitive response. Future work could analyze the effectiveness of time-sensitive notifications versus basic notifications. However, even time-sensitive notifications could be delayed or ignored based on the user’s settings. We recommend that mobile sensing researchers review notification settings during onboarding. Persistent reminders offer an additional way to remind users to complete tasks. Seven participants (6 iOS and 1 Android) reported that the persistent reminder helped them to remember to complete their assessment and could be an area to explore further.

Besides notification deferral, notification summaries, and contextual prompting, other notification system advances can potentially disrupt notification-initiated EMA. Because notifications disrupt users, sometimes causing stress [42-44], several modifications to notification systems could be introduced to consumer mobile devices. Lin et al [45] demonstrated how notification summaries could be improved by letting users determine the order of the notifications. Pejovic et al [46] explored user contexts to understand user interruptability, leading to the development of intelligent notification systems [34,47]. Kandappu et al [48] also explored intelligently interrupting users. Mobile sensing apps must adjust to these advanced notification systems or find a different way to initiate EMA. For example, Zhang et al [49] explored using a persistent reminder on the lock screen to initiate EMA.

### Event-Driven Passive Data Collection

For event-driven data collection, use carefully designed contextual prompts. Our qualitative analysis revealed that most participants were willing to authorize background location access for research studies and were under the impression that they enabled background location access. However, our quantitative results revealed that many participants did not authorize background location access. Many failed to grant authorization for background events even though they intended to authorize it. As we hypothesized, contextual prompting on iOS was 55.5% more effective in gaining authorization for background location events. This coincides with previous work that shows additional context helps users make better privacy decisions [39,50,51].

Even with contextual prompts, less than half of the participants authorized access to background location events. Challenges arise when users misinterpret authorization prompts, limiting the success of event-based collection [39,41,51-53]. To improve contextual prompt accuracy, Wijesekera et al [40] developed a machine learning–based, contextual-aware permission model that improves the permission accuracy rate above context prompts, which should be considered if it becomes available on consumer mobile devices. However, the current model suggests providing “generic but well-formed data” when an app is denied access. In the context of health sensing apps, this can skew results and create adverse health interventions. We recommend that such systems be designed to communicate to the app about denials and that apps be constructed to handle denials to ensure data fidelity. Further research must be performed to balance the collection abilities of mobile sensing apps and protect the privacy and security of their users [54].

### Polling-Based Passive Data Collection

For polling, implement a persistent reminder that can be used both as a means of collecting data and as a reminder to complete EMA tasks. Collecting data through background tasks presented significant challenges due to the use of an implied consent model for granting background runtime that is inherent to mobile OS, as described in previous work [36,55]. Although Android devices are generally more permissive, too many factors are under consideration to consistently guarantee background runtime. However, as we hypothesized, using a persistent reminder as a secondary means to poll for data yielded more successful data collection. Installing a persistent reminder on the user’s home screen signifies to the OS the intent to allocate the necessary resources to maintain the reminder’s regular updates, which can also be used for passive data collection.

Many users did not comply with installing the widget when prompted by the app, especially among Android users. Verbal prompts to install the widget did help to improve compliance on Android and iOS. Our qualitative analysis indicated some resistance to installing the widget because users did not want to change their home screen layout, and some were unfamiliar with how to install widgets or did not use widgets.

Several methods are available to overcome the resistance to using widgets. First, participants who more directly benefit from the data collection might be more willing to install a widget. For instance, participants affected by a disease might be more willing to install a widget, especially if the app offers just-in-time, adaptive interventions. In addition, study incentives can also be increased for participants who install the widget. To assist users unfamiliar with widgets, the app can provide a video tutorial on installing the widget, or instructions can be provided during study onboarding.

### Limitations

There are other differences between iOS and Android devices and users of those devices than what we tested for, which could be confounding factors. In addition, we studied passive sensing for research studies, and the results do not necessarily apply to passive sensing in other contexts such as crowdsourcing or commercial purposes.

This study involved college students, and while they represented diverse academic disciplines, races, and countries of origin, they were all aged 19-26 years and tended to be more familiar with mobile devices. Further work should be conducted to observe how these results would translate to a larger, more heterogeneous population. Individuals less familiar with mobile devices, such as geriatric patients, may exhibit more difficulty granting and declining authorization due to different privacy preferences or familiarity with smartphone technology.

For persistent reminders, only two Android users installed the widget, with one device refreshing more than once per day and the other refreshing 23 of the 30 days. These preliminary results are promising. However, further work is required to ensure the results are consistent with a larger sample size.

### Future Work

Additional modifications to the mobile apps could be implemented and tested in follow-up studies. Although Android does not allow contextual prompts, contextual prompts could be mimicked by randomly sending a notification asking for background location access or notification access. Such a study would eliminate potential confounding factors that could have influenced the results.

Our sensing apps could be used in additional health studies incorporating other populations with different motivations to comply with study procedures. Comparing our results with studies involving more diverse health issues would be an interesting avenue for future work.

### Comparison With Prior Work

Numerous studies have contributed to a comprehensive understanding of EMA compliance across research fields [56]. In a review of EMA studies, Wrzus and Neubauer [57] found that compliance cannot be predicted by the number of assessments or length of the study but that financial incentives did improve compliance rates. Murray et al [58] explored the role of participants’ emotional states in affecting compliance. In the related field of crowdsourcing [59], gamification can improve response rates [60]. Other efforts to improve EMA compliance include the work by Schenider et al [61] on just-in-time, adaptive EMA to reduce the burden on participants. Zhang et al [49] explored unlock journaling, where users unlock their devices by completing an EMA. This work built upon this by examining how notification permissions affected EMA compliance.

Mobile sensing depends upon gaining proper authorization to collect data and interrupting participants to initiate an EMA. Research into permissions on mobile devices has shown that users often misinterpret permission prompts [52,53]. Wijesekera et al [39,40] showed that contextual prompts help users correctly interpret permission prompts. Alsoubai et al [41] profiled users to help understand differing privacy strategies, which helps improve intelligent permission systems [40]. Our mobile sensing apps explored contextual prompts and permissions and their role in passive data collection.

Prior work has demonstrated that collecting consistent data across iOS and Android devices remains challenging [62]. Most mobile sensing studies use Android devices due to their more open programming interface, but some work has been conducted to improve mobile sensing on iOS devices. The AWARE-iOS research team [55] explored background data collection methods on iOS and developed guidelines for sustainable data collection on iOS. AWARE-iOS has successfully collected passive data on iOS devices [63]. RADAR-base [64,65] is an open-source mobile health platform for collecting and analyzing large-scale data from various devices including passive and active mobile apps. We expand upon this inspirational prior work by adding design guidelines for consistent active and passive data collection across Android and iOS devices. Consistent data collection is needed to support just-in-time adaptive interventions on consumer mobile devices, necessitating further research [66].

### Conclusions

We developed and tested mobile sensing apps for iOS and Android to answer our RQs: (RQ1) How do contextual prompting and setup prompting affect scheduled notification delivery and the response rate of notification-initiated EMA? (RQ2) Which authorization paradigm, setup or contextual prompting, is more successful in leading users to grant authorization to receive background events? and (RQ3) Which polling-based method, persistent reminders or scheduled background tasks, completes more background sessions? Although contextual prompts for notification authorization on iOS devices did not impact EMA compliance rates compared to setup prompts on Android devices, many iOS users reported not receiving notifications. For background event authorization, contextual prompts on iOS devices were 55.5% more effective in gaining authorization than setup prompts on Android devices. Finally, persistent reminders resulted in a completion of background sessions 266.5% more often than when using traditional background tasks. However, we observed some user resistance to installing persistent reminders. Although mobile sensing on consumer mobile devices continues to exhibit challenges, our results suggest that persistent reminders and proper authorization procedures can improve user compliance.

## Appendix

Multimedia Appendix 1 iCHECK Checklist for Digital Health Implementations.

## Abbreviations

EMA: ecological momentary assessment
OS: operating system
RQ: research question
UI: user interface

## Data Availability

The datasets generated during or analyzed during this study are not publicly available due to institutional review board restrictions but are available from the first author upon reasonable request.
